# Supplementary figures and images for: GAMBIT (Genomic Approximation Method for Bacterial Identification and Tracking): A methodology to rapidly leverage whole genome sequencing of bacterial isolates for clinical identification
Source: PLoS One. 2023 Feb 16;18(2):e0277575. doi: 10.1371/journal.pone.0277575 (PMC9934365; doi:10.1371/journal.pone.0277575)

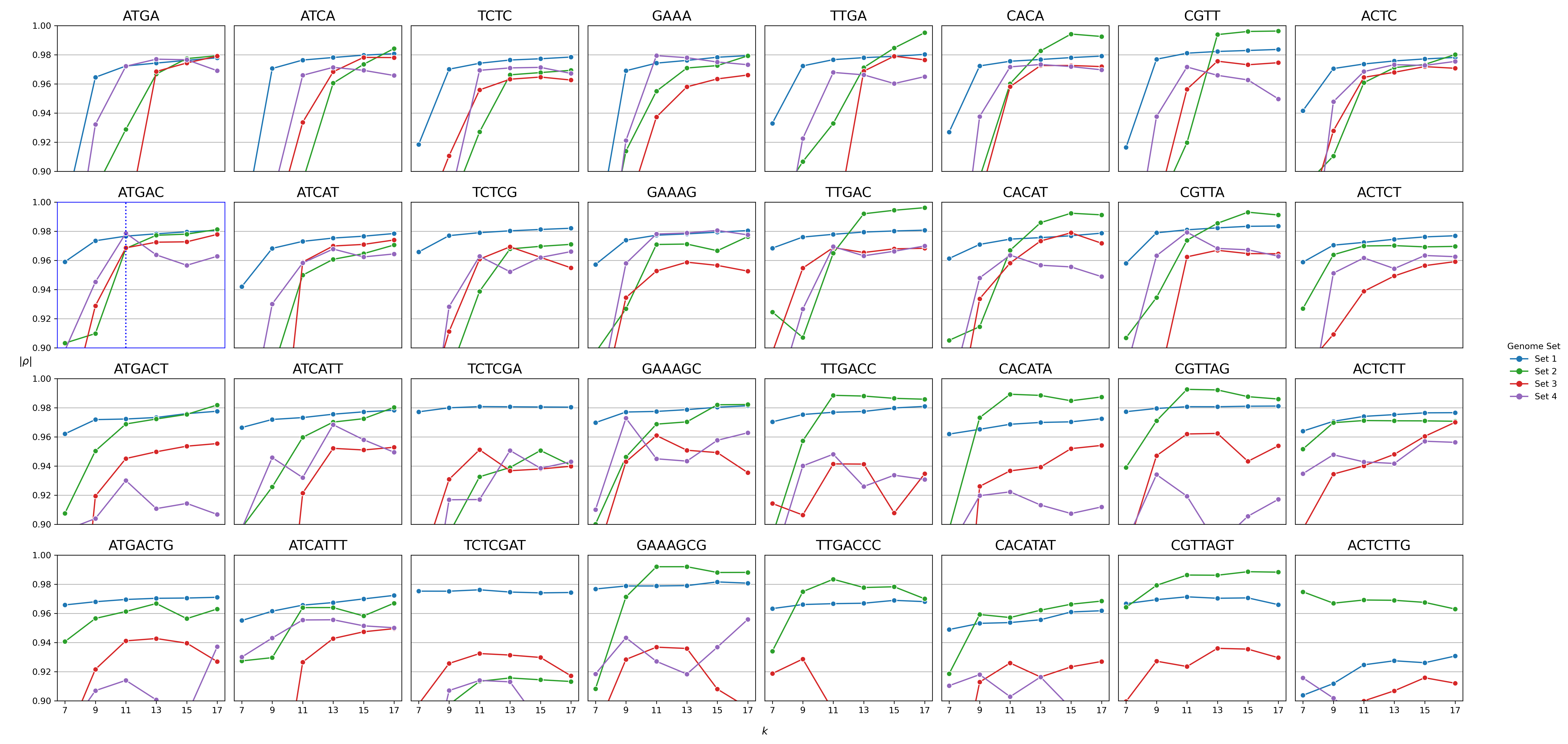

Supplement: S1 Fig — Subplots represent different choices of prefix sequence for the GAMBIT distance metric. Columns correspond to eight 7-mer base sequences (our default ATGAC plus two random nucleotides and seven fully random sequences). Rows correspond to truncations of these base sequences to 4, 5, 6, and 7 nucleotides. As in Fig 2 each subplot shows the absolute value of the Spearman correlation between GAMBIT distance and ANI vs value of the k parameter for all genome pairs in data sets 1–4. (PNG) [file pone.0277575.s001.png]

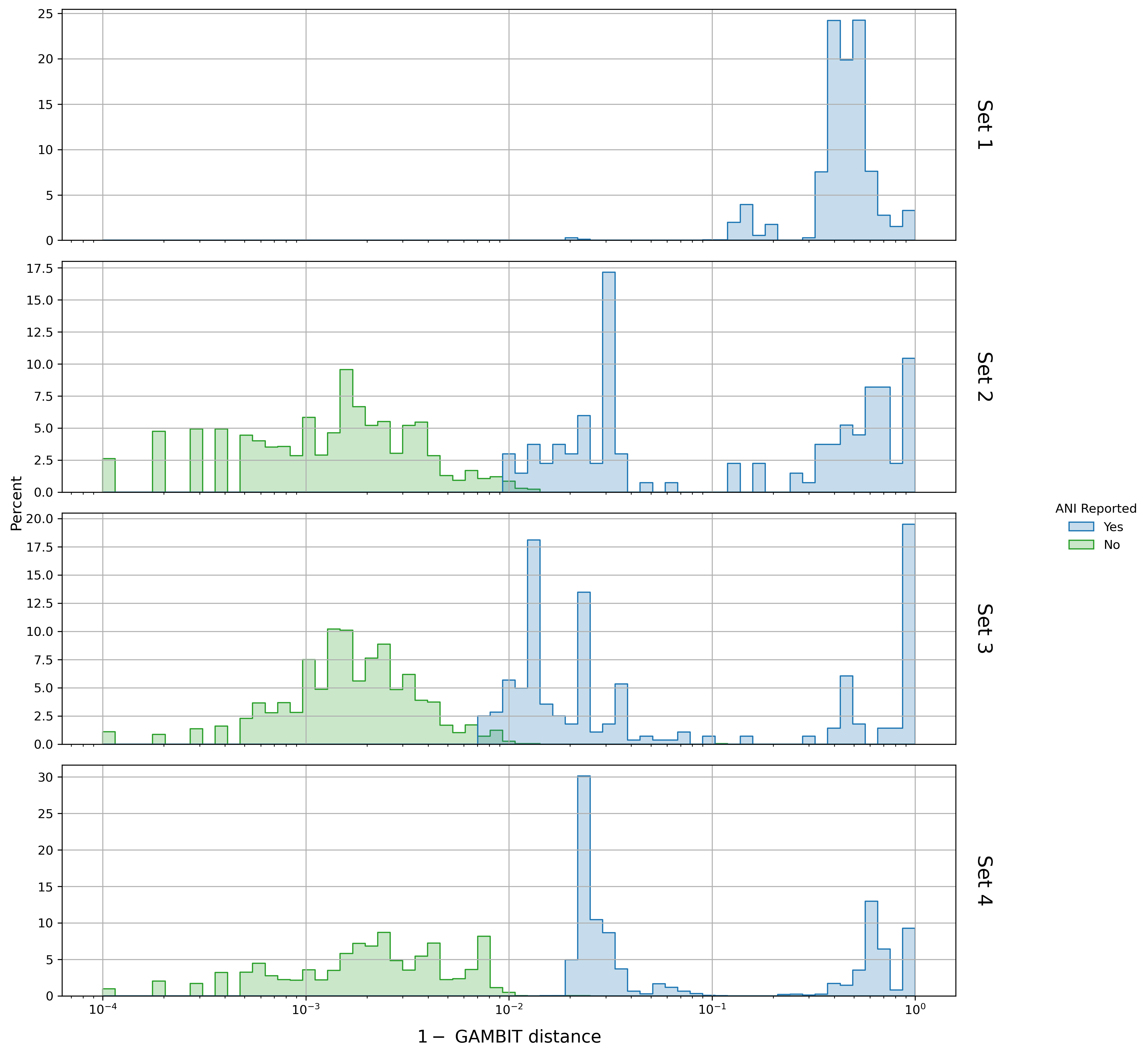

Supplement: S2 Fig — Shown are distributions of GAMBIT similarity values (one minus the GAMBIT distance) for all pairs of genomes in sets 1–4, split based on whether the FastANI tool did (blue) or did not (green) report an ANI value for the pair. FastANI’s cutoff of approximately 80% corresponds to a GAMBIT distance of approximately 0.99 (similarity 0.01), with very little overlap between the two groups. Note that FastANI reported a value for all pairs in set 1. (PNG) [file pone.0277575.s002.png]
