# Supplementary material for: GAMBIT (Genomic Approximation Method for Bacterial Identification and Tracking): A methodology to rapidly leverage whole genome sequencing of bacterial isolates for clinical identification
Source: PLoS One. 2023 Feb 16;18(2):e0277575. doi: 10.1371/journal.pone.0277575 (PMC9934365; doi:10.1371/journal.pone.0277575)
Supplement: Attachment — Submitted filename: response to reviewers v2.docx [file pone.0277575.s012.docx]

Response to Reviewers

Below are the changes we made to the latest revised manuscript of GAMBIT (Genomic Approximation Method for Bacterial Identification and Tracking): A methodology to rapidly leverage whole genome sequencing of bacterial isolates for clinical identification”.

Reviewer #2: I appreciate the authors attention to detail in making the necessary corrections to the manuscript, including my rather passionate defense of clinical microbiology. I would only ask for a few minor changes to that portion of the response.

Line 570 - change "trade" to "profession" or even "work" - although it should not carry a derogatory connotation, trade in this instance seems dismissive.

“trade” was changed to “profession”

Line 572 - "more quickly" - i'm not sure this claim is backed up by the data presented and no data is cited here to back up this claim. Given the time involved in WGS sample preparation, sequencing, and bioinformatics analysis compared
to routine lab workflow. Would strike unless this can be supported.

Stuck the works “trained more quickly, and” the sentence now reads

“While WGS cannot replace this, it can provide a comprehensive means of identification that can be performed systematically.”

Line 574 - simply doing the sequencing doesn't realize these potential benefits... there is additional downstream work that has to be done post sequencing... so I would qualify as "can be realized"

Changed “realized” to “can be realized”
